# Microsecond-Scale Molecular Dynamics Simulation of Phase Transition of a Bilayer Ice: Kinetic Constraints in Confined Water

**DOI:** 10.1021/acs.jpcb.5c01346

**Published:** 2025-06-09

**Authors:** Weiduo Zhu, Yiyao Li, Haidi Wang, Zhao Chen, Xiaofeng Liu, Zhongjun Li, Wenhui Zhao, Xiao Cheng Zeng

**Affiliations:** † Department of Physics, 12513 Hefei University of Technology, Hefei, Anhui 230009, China; ‡ Department of Physics, 47862 Ningbo University, Ningbo, Zhejiang 315211, China; § Department of Materials Science & Engineering, 53025 City University of Hong Kong, Kowloon, Hong Kong 999077, China

## Abstract

In this study, we investigate the phase behavior of water confined between two parallel smooth walls by using both classical molecular dynamics (MD) simulations and machine-learned potential (MLP) MD simulations. Particular attention is focused toward the water-to-ice phase transition below the freezing point. Three distinct two-dimensional (2D) bilayer (BL) crystalline ice phases are observed, namely, bilayer hexagonal ice (BL-ice I), bilayer very high-density ice (BL-VHDI), and a newly found bilayer penta-hexa ice (BL-PHI). The latter consists of interlocked pentagonal and hexagonal rings. The transition from liquid to BL-PHI is weakly first-order, and typically, the BL-PHI emerges at intermediate to high lateral pressures (400 to 900 MPa) after microsecond-scale simulations, highlighting its relatively slow formation process. Compared to BL-ice I and BL-VHDI, BL-PHI exhibits much higher diffusion activation energy and hence a much slower freezing rate. Additionally, the transition temperatures of all three bilayer ices are pressure-dependent. These findings provide new insights into the complex behavior of nanoconfined water.

## Graphical abstract

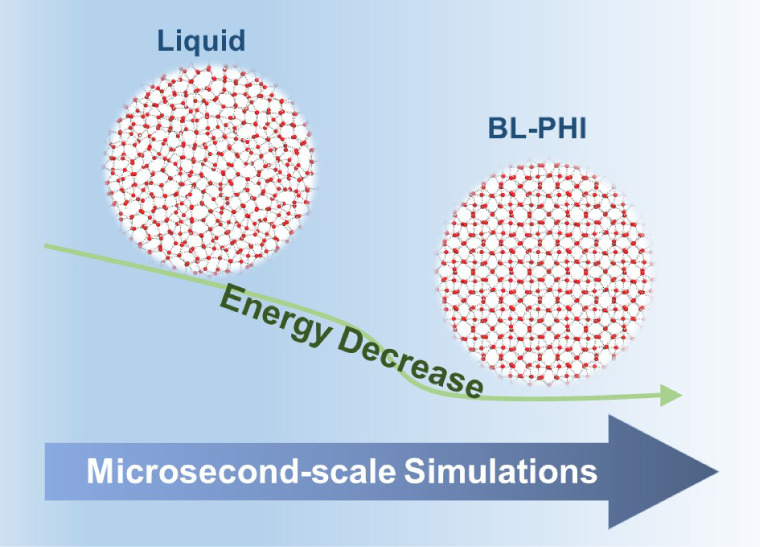

## Introduction

Water is a vital substance with extraordinary physicochemical properties that shape Earth’s environment and sustain life. The phase diagram of bulk water features at least 20 crystalline ice phases under varying temperature and pressure conditions.^1–4^ Under nanoscale confinement, water’s phase behavior becomes even more complex due to the interplay between intermolecular hydrogen bonding and spatial restrictions of nanoconfinement. This interplay leads to novel crystalline structures and phase transitions not observed in bulk water. For example, water in angstrom-scale slits can form various two-dimensional (2D) monolayer ices, including monolayer hexagonal ice, monolayer flat rhombic ice, monolayer puckered rhombic ice, and high-density monolayer square ice.^5–8^ In slightly wider nanoslits, diverse bilayer ice structures can emerge, such as bilayer hexagonal ice (BL-ice I),^9,10^ bilayer interlocked pentagonal ice,^11^ bilayer triangular ice,^12,13^ bilayer low-density and very high-density amorphous ice,^14,15^ bilayer very high-density ice (BL-VHDI),^15,16^ and the recently reported bilayer partially ionic ice and superionic ice.^17^

These complex phase behaviors of nanoconfined water are particularly evident during phase transitions, especially the liquid-to-solid freezing transition. Understanding these transitions is crucial for numerous environmental, biological, and industrial applications. The nature of these transitions, whether first-order or continuous, depends on the specific conditions and the ice structures formed spontaneously. Numerous classical MD studies have documented a wide range of phase transitions, such as liquid–solid,^14,18^ liquid–liquid,^19^ solid–solid,^20,21^ and solid–liquid–solid transformations,^10^ among others. These transitions are mostly first order, while some are continuous.^22–24^

Recently, machine learning potentials (MLPs) have been increasingly used in large-scale molecular dynamics simulations with near-first-principles accuracy and significantly enhanced computational efficiency. For instance, Kapil et al. employed both quantum Monte Carlo (QMC) methods and MLP-based MD simulations to construct a detailed pressure–temperature phase diagram of monolayer water confined within graphene-like nanochannels. Their study identified multiple monolayer ice phases, such as a novel superionic phase, and a continuous phase transition between a hexatic-like monolayer ice and the liquid phase.^25^ Similarly, Lin et al. examined the phase behavior of monolayer water and ice confined in a 6.0 Å nanoslit, predicting a diverse array of structures, including various monolayer ice structures and quasi-bilayer ice (qBI) phases, along with both first-order and continuous phase transitions.^26^ Later, Jiang et al. developed an MLP and employed large-scale path-integral molecular dynamics simulations to investigate proton dynamics and phase behavior of monolayer and bilayer ice under nanoconfinement and high pressures.^17^ They predicted 10 distinct two-dimensional ice phases and characterized a unique solid-melting process involving successive double or triple continuous transitions—from bilayer molecular ice to plastic ice, then to hexatic ice, and eventually to a superionic fluid. Collectively, these studies provide new insights into the complex phase behavior of nanoconfined water and offer valuable theoretical guidance for the future experimental realization of two-dimensional ice phases.

Despite significant progress, the structure and phase behavior of water confined within angstrom-scale slits remain incompletely known due to the complex interplay of hydrogen bonding, water-confinement wall interactions, and confinement effects. In this work, we focus on the phase behavior of water confined in hydrophobic angstrom-scale slits. Using MD simulations, we show that depending on the lateral pressures, water can freeze into distinct crystalline structures via first-order phase transitions. At a nanoconfinement width of 8.6 Å, three bilayer crystalline phases are observed, namely, BL-ice I, BL-VHDI, and bilayer penta-hexa ice (BL-PHI). As a new 2D ice, BL-PHI—comprising interlocked pentagonal and hexagonal rings—emerges at intermediate to high pressures. Furthermore, the MLP-based MD simulations demonstrate that the BL-PHI phase maintains its structural integrity over a wide pressure range (400–900 MPa) for at least 5 ns at 150 K, indicating its thermodynamic stability. Notably, unlike other bilayer ice formations, the freezing transition from liquid water to BL-PHI is a weak first-order transition that requires microsecond-scale simulations to form. Further analysis of diffusion coefficients and activation energies shows that BL-PHI exhibits a significantly higher diffusion activation energy before ice formation compared to BL-ice I and BL-VHDI. This relatively high activation energy hinders molecular diffusion, thereby slowing down the freezing process. Our findings explain why BL-PHI was not reported previously and provided new molecular insights into the phase behavior and dynamics of nanoconfined water.

## Models and Methods

We perform a series of classical molecular dynamics (MD) simulations of water confined between two smooth surfaces, separated by a fixed distance of 8.6 Å, by using the GROMACS software package. The simulation system is similar to those used in previous studies.^10,11^ It consists of 792 water molecules with intermolecular interactions modeled by the TIP4*P*/2005 pair potential.^27^ The model accurately captures key thermodynamic properties of liquid water and ice phases, including relative energies, critical temperature, and surface tension, and reproduces the realistic temperature–pressure (*T*–*P*) phase diagram. It also resolves well-known structural motifs of ice polymorphs, such as ice Ih, II, III, V, VI, and VII.^28,29^ Additionally, parallel simulations using the TIP4P/Ice force field yield consistent results (Table S1), demonstrating the robustness of our findings. Water interaction with the smooth hydrophobic surfaces is described by the 9-3 Lennard-Jones (L-J) potential with parameters of σ_o‑wl_ = 0.25 nm and ε_o‑wl_ = 1.25 kJ/mol.^30–33^

All MD simulations are performed in the isothermal/isolateral-pressure (*NP*_L_ *T*) ensemble, with periodic boundary conditions applied in the lateral (*x* and *y*) directions. Temperature (*T*) and lateral pressure (*P*_L_) are controlled by using the Nosé–Hoover thermostat^34,35^ and the Parrinello–Rahman barostat,^36^ respectively. A cutoff of 1.2 nm is adopted for the L-J pair interactions, and the long-range electrostatic interactions are treated using the slab-adapted Ewald sum method.^37^ To search for the phase transitions of confined water, we simulate both the cooling and heating processes. The temperature is varied with an interval of 10 K, and lateral pressure is set from 0.1 MPa to 1.5 GPa, with an interval of 50 MPa. For given *P*_L_ and T, an equilibration run is performed for 20–1100 ns, with a time step of 2 fs.

## Results and Discussion

The confined water is first equilibrated at 300 K under various lateral pressures. The temperature is then reduced from 300 to 180 K in steps of 10 K, resulting in the emergence of three distinct crystalline structures, each dependent on the applied lateral pressure (Figure 1 and S1). Notably, at medium to high pressures (400 to 900 MPa), a novel bilayer ice structure, named BL-PHI, is observed after conducting simulations on a microsecond scale (Figure 1). This long time scale is essential for the formation of BL-PHI, as it allows the phase transition from liquid to occur and reveals the unique characteristics of this new phase. Although initially appearing amorphous (Figure 1a), further examination, as discussed below, reveals that BL-PHI exhibits distinct crystalline characteristics. This unique phase features both pentagonal and hexagonal rings with dislocations present in the water molecules within each layer (Figure 1c–d). A closer look at the monolayer top view of BL-PHI shows a nearly uniform mixture of pentagonal and hexagonal rings, with the ice rule being satisfied, as observed in most BL crystalline ices reported thus far, although some defects are evident. From the side view, the water molecules within each layer are not perfectly aligned within the same plane, instead exhibiting slight undulations in the vertical direction (Figure 1b). These structural irregularities indicate a more complex arrangement than was initially expected, distinguishing BL-PHI from the other phases.

**Figure 1:**
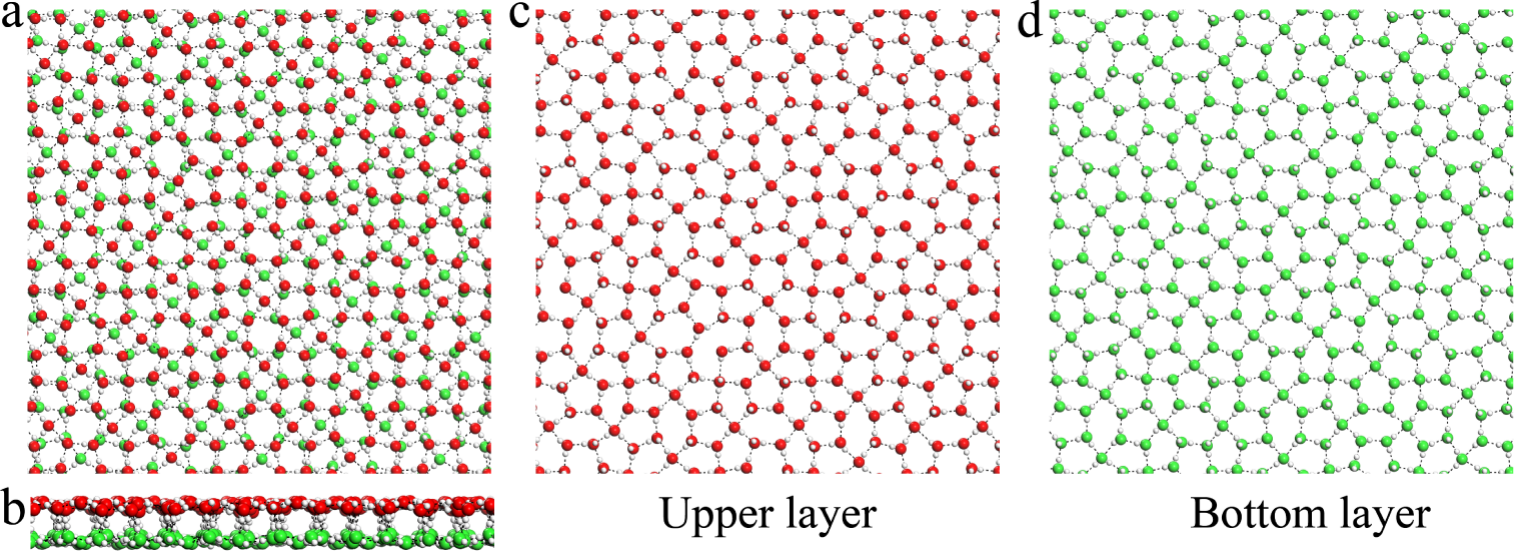
(a) Top view and (b) side view of snapshots depicting the inherent structures of the BL-PHI at 200 K and 400 MPa confined between two hydrophobic walls with a separation distance of *h* = 8.6 Å. (c) Top view of the BL-PHI highlighting the upper monolayer in red and (d) the bottom monolayer in green. Oxygen atoms are shown as red and green spheres, hydrogen atoms as white spheres, and hydrogen bonds by black dashed lines.

In contrast, at lower pressures (ranging from 0.1 to 350 MPa), bilayer liquid water spontaneously transforms into BL-ice I as the temperature decreases (Figure S1a). This phase, characterized by hexagonal rings, is well documented in previous studies and serves as a baseline for understanding water’s behavior in nanoconfinement.^9,34^ BL-ice I undergoes this transition on a nanosecond time scale, which is significantly faster than the microsecond-scale transition required for BL-PHI. Further increasing the lateral pressure (1.0 to 1.5 GPa) results in the formation of BL-VHDI (Figure S1b), as reported in our previous study.^10^ This phase not only shows an increase in density but also exhibits unique lattice characteristics, where the water molecules are arranged in a compact and highly ordered manner, forming an array of square ice nanotubes. The notable difference in formation times between BL-PHI (which requires microsecond-scale simulations) and both BL-ice I and BL-VHDI (which form within nanoseconds) highlights the distinctive and slower formation process of the BL-PHI phase.

Figure 2 illustrates the potential energy (*U*) per water molecule versus temperature (*T*) under varying lateral pressures (*P*_L_), highlighting the occurrence of phase transitions. At relatively low pressures (0.1–350 MPa), the potential energy decreases progressively with cooling before undergoing an abrupt decline of 2.0–4.0 kJ/mol, a hallmark of first-order phase transitions from liquid water to BL-ice I. The specific temperature range for the transitions depends on the applied lateral pressure, decreasing from 270 K at 0.1 MPa to 200 K at 350 MPa (Figure 2a).

**Figure 2:**
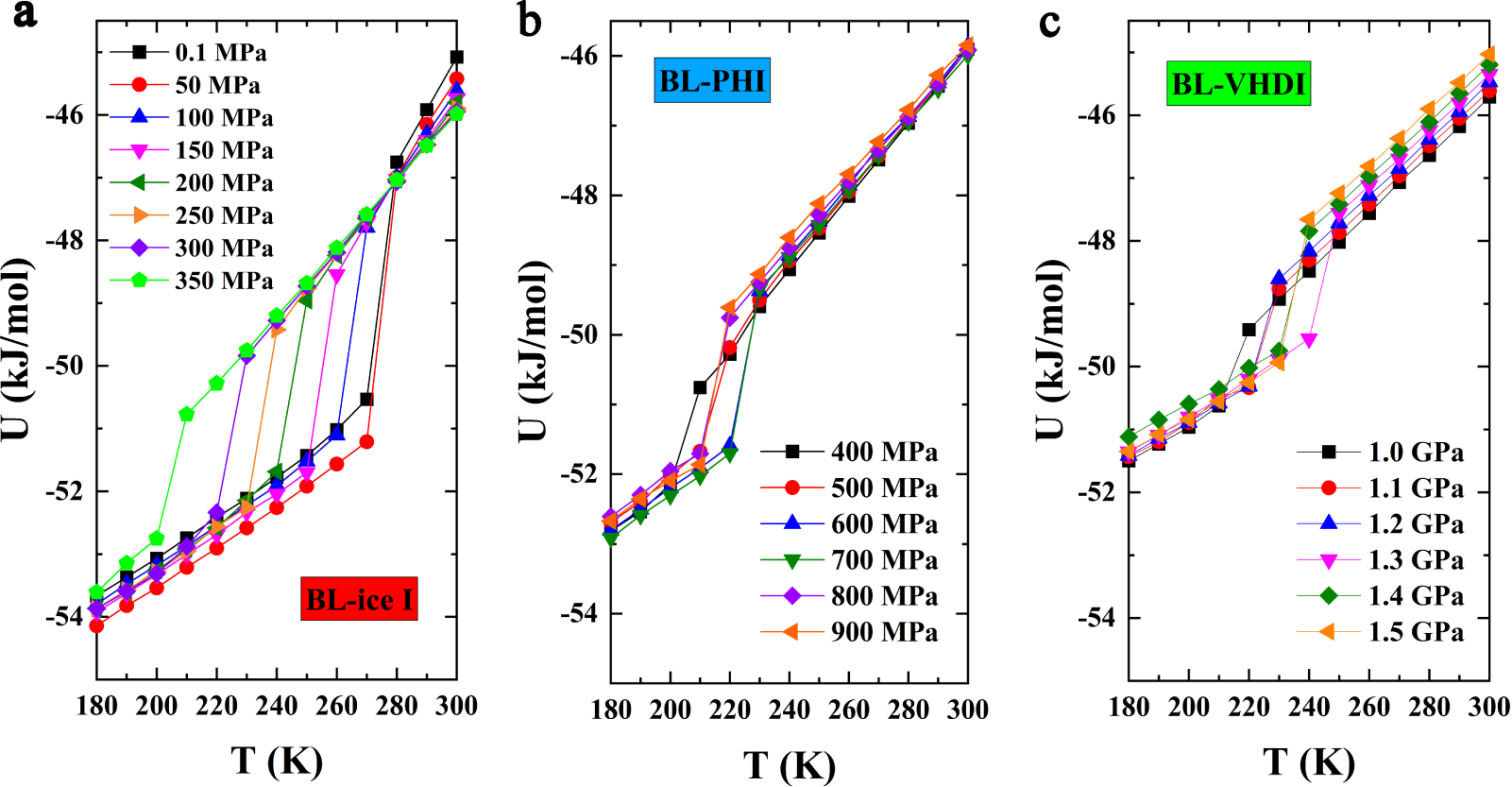
Variation of potential energy (*U*) with temperature (*T*) during the cooling process at different pressures. (a) Lateral pressure (*P*_*L*_) from 0.1 to 350 MPa for BL-ice I. (b) Lateral pressure (*P*_*L*_) from 400 to 900 MPa for BL-PHI. (c) Lateral pressure (*P*_*L*_) from 1.0 to 1.5 GPa for BL-VHDI.

The phase transition from liquid water to the BL-PHI occurs in the medium-to-high-pressure regime (400–900 MPa), where sudden potential energy drops of approximately 1.5–2.0 kJ/mol indicate the first-order phase transitions. Interestingly, the BL-PHI transition temperature exhibits a nonmonotonic pressure dependence: it initially rises from 200 K at 400 MPa to a maximum of 220 K at 600–700 MPa and then decreases to 210 K at 800–900 MPa. This nonmonotonic variation in the temperature–pressure relationship suggests certain competing structural properties of the BL-PHI.

At high pressures (>1.0 GPa), the freezing transition into the BL-VHDI phase is characterized by the smaller potential energy drop (0.5–1.5 kJ/mol), suggesting weakly first-order behavior. The BL-VHDI transition temperature first increases from 210 K at 1.0 GPa to 240 K at 1.3 GPa and then modestly declines to 230 K at 1.4–1.5 GPa. The irreversibility of these transitions due to thermal hysteresis, evident in heating–cooling cycles (Figure S2), further corroborates their first-order nature. The BL-PHI phase can be viewed as an intermediate state bridging low-pressure and ultrahigh-pressure regimes, with its distinct temperature–pressure phase behavior underscoring unique structural reorganization mechanisms under confinement.

To elucidate the structural distinctions among the three bilayer ice phases, we analyze the transverse density profile (TDP) of hydrogen and oxygen atoms along the *z*-axis perpendicular to the walls, with particular emphasis on the BL-PHI phase. Unlike BL-ice I and BL-VHDI, where hydrogen TDPs exhibit a characteristic 3:1 ratio between higher and lower peaks (reflecting three intralayer hydrogen bonds and one interlayer hydrogen bond per molecule), the BL-PHI phase shows a markedly reduced lower hydrogen peak (Figure 3a). This attenuation directly correlates with a subset of water molecules in BL-PHI forming four fully intralayer hydrogen bonds, effectively eliminating interlayer connectivity—a stark contrast to the other two phases. Additionally, the TDP for oxygen atoms in BL-PHI exhibits a shoulder in both peaks, suggesting that water molecules are slightly staggered rather than lying flat, a deviation not seen in BL-ice I and BL-VHDI. To maintain the tetrahedral hydrogen-bond network, water molecules forming tetrahydrogen bonds within the layer must slightly offset along the *z*-direction.

**Figure 3:**
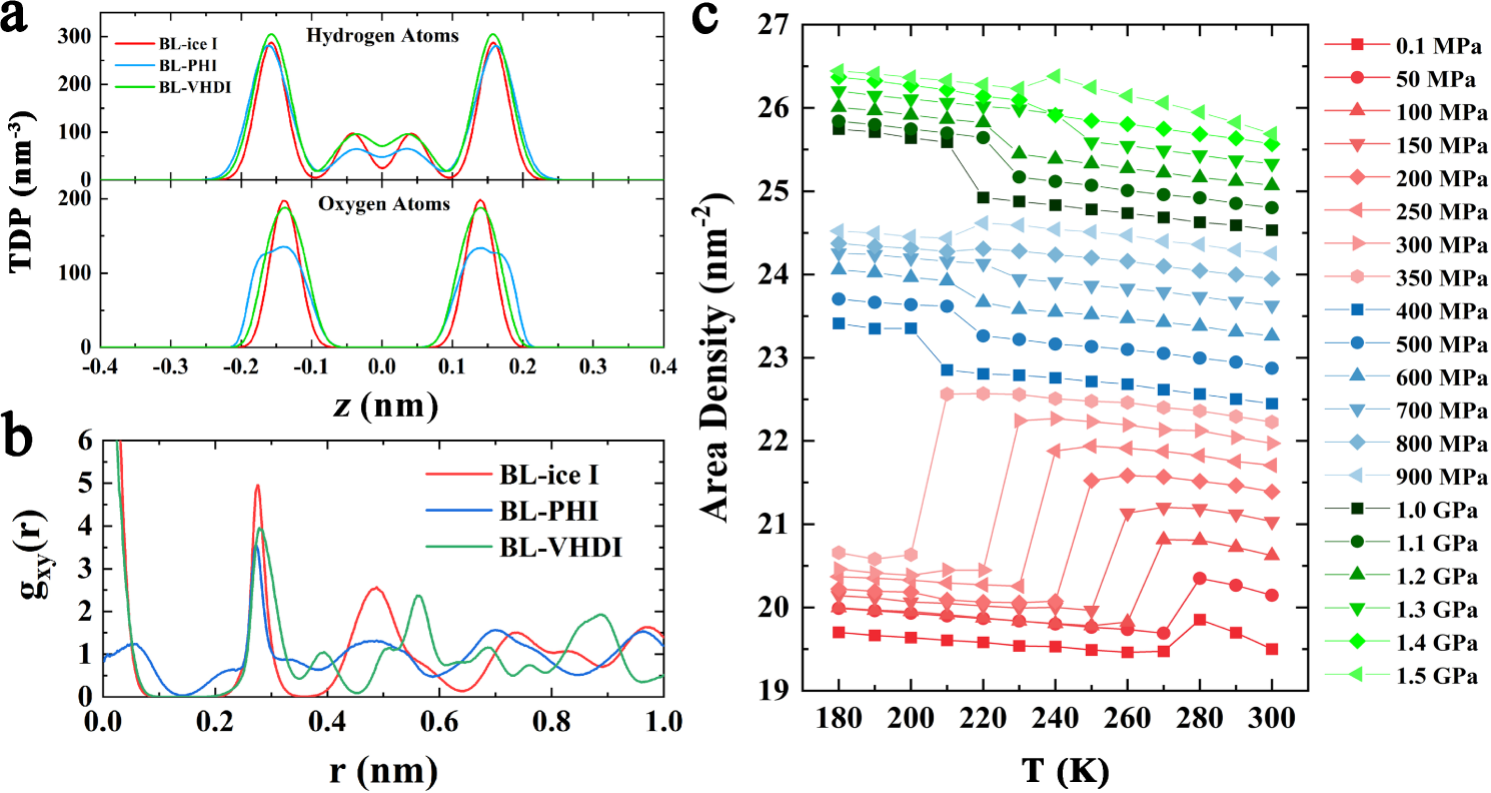
Structural characteristics of the bilayer ices. (a) Transverse density profile (TDP) of nanoconfined ices at 200 K. (b) The lateral oxygen–oxygen radial distribution functions (RDFs) at 200 K. (c) Area density versus temperature (*T*) for the cooling system.

Further structural insights into BL-PHI are obtained through lateral oxygen–oxygen radial distribution functions (RDFs). As shown in Figure 3b, the well-defined peaks and valleys in the RDFs confirm the long-range crystalline order in bilayer ices. Unlike BL-ice I and BL-VHDI, for which a prominent peak at ∼0.0 Å signifies registry-aligned oxygen atoms between layers (AA stacking), the BL-PHI displays a small peak at ∼0.6 Å. This offset reflects a staggered interlayer oxygen arrangement, a structural feature of BL-PHI. To gain deeper insight into this stacking configuration, the lateral RDFs of the upper and lower monolayers were separately calculated to analyze their stacking configuration. As shown in Figure S3, the lateral RDFs of the upper and lower layers almost overlap, indicating that the two monolayers of the bilayer ice have similar structures. For BL-ice I and BL-VHDI, bilayer RDFs nearly replicate monolayer profiles, consistent with perfect AA stacking. In contrast, BL-PHI’s bilayer RDF differs markedly from its monolayer counterpart at short distances, evidencing nonregistry stacking where upper and lower layers shift laterally relative to each other.

Density changes during phase transition further distinguish BL-PHI. As shown in Figure 3c, abrupt changes in the area density mark the transitions between different bilayer ice phases. BL-PHI exhibits area densities ranging from 23.4 nm^–2^ to 24.5 nm^–2^, positioning it between the lower-density BL-ice I (19.7–20.8 nm^–2^) and the higher-density BL-VHDI (25.7–26.5 nm^–2^). This intermediate density range reflects BL-PHI’s unique hydrogen-bonding network, which includes both pentagonal and hexagonal motifs, leading to a denser packing than BL-ice I but a more open structure than BL-VHDI. Notably, at pressures relevant to BL-ice I, bilayer liquid water spontaneously freezes into BL-ice I, accompanied by a decrease in system density, similar to the freezing behavior of bulk water. At higher pressures relevant to BL-PHI, either low-density or high-density liquid water can freeze into bilayer ices of intermediate density. In other words, during the formation of BL-PHI, the density change is pressure-dependent: between 400 and 700 MPa, freezing of the liquid water increases density, while at 800–900 MPa, freezing leads to a decrease in density. A similar trend is observed for BL-VHDI. This behavior aligns with the change in phase-transition temperature for BL-PHI and BL-VHDI (initial increases followed by decreases).

To compare the nucleation kinetics for the three types of bilayer ice, we select representative pressures (250 MPa, 400 MPa, and 1.2 GPa) with minimal structural defects during spontaneous freezing. As shown in Figure 4a–c, BL-ice I undergoes a liquid–solid phase transition at 230 K and 250 MPa. The potential energy begins to decrease after approximately 2 ns of simulation, and the freezing process is complete at around 10 ns, at which point the potential energy stabilizes. For BL-VHDI, the phase transition occurs at 220 K and 1.2 GPa. The potential energy begins to decrease after about 35 ns and reached equilibrium after 55 ns.

**Figure 4:**
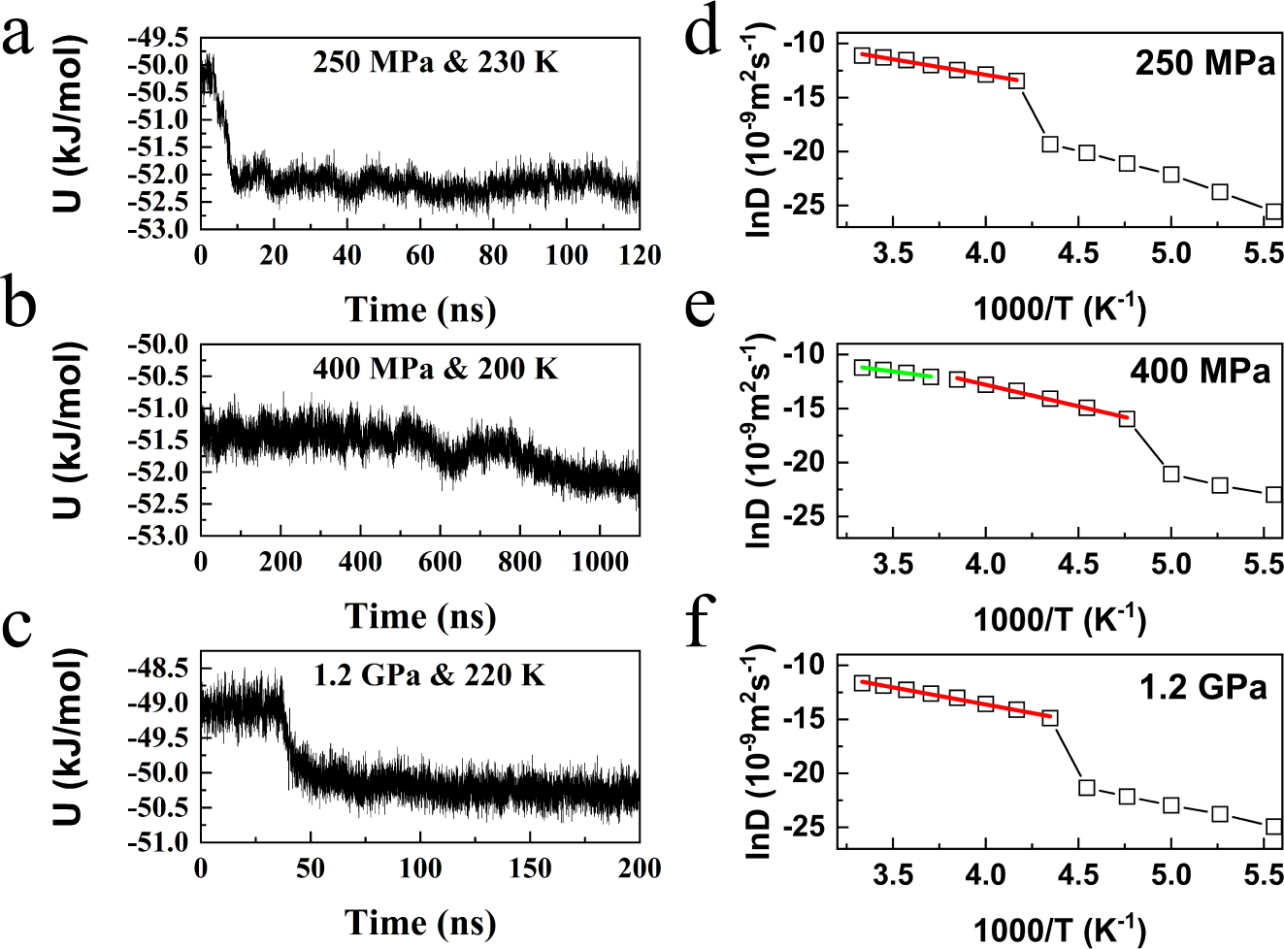
Results of MD simulations to identify the freezing transition from liquid water to bilayer ice. (a–c) Time-dependent potential energy (per water molecule) of the system during the MD simulation at different lateral pressures (*P_L_*) and temperatures (*T*). (e-f) Computed diffusion coefficient versus pressure and reciprocal temperature in Figure 3a–c. The temperature dependence can be fitted with an Arrhenius law as red and green lines indicate.

In contrast, for BL-PHI, the phase transition at 200 K and 400 MPa occurs on a much longer time scale. Freezing begins only after a 500 ns simulation, with the potential energy gradually decreasing over 1000 ns before stabilizing. This indicates that the transition from liquid to BL-PHI involves a much higher nucleation barrier, resulting in a significantly slower nucleation rate compared to BL-ice I and BL-VHDI. The prolonged nucleation process likely arises from BL-PHI’s metastable hybrid configuration, which combines staggered interlayer stacking with an intermediate density (23.4–24.5 nm^–2^). This unique structural arrangement demands precise molecular reorientation to maintain a balance between intralayer hydrogen-bonding networks and interlayer registry mismatches. The resulting geometric frustration suppresses the formation of a critical nucleus, contrary to the more straightforward AA-stacking pathways that facilitate the rapid nucleation and crystallization of BL-ice I and BL-VHDI.

To further investigate the nucleation and growth process, we analyze the diffusion coefficients of the three bilayer ice phases at different temperatures. These diffusion coefficients are computed from the mean square displacement (MSD) parallel to the confining planes in equilibrium *NP*_L_ *T* simulations, as described by *x*(*t*)^2^ + *y*(*t*)^2^ = 4*Dt* (Figure 4–f). At relatively high temperatures, the lateral MSD increases linearly with time, indicating a liquid phase, whereas at lower temperatures, the MSD exhibits oscillations with minimal slopes characteristic of a solid phase (Figure S4). The abrupt changes in diffusion coefficients suggest that the liquid–solid phase transitions for all three ices are first order, consistent with the potential energy variations observed earlier.

According to the Arrhenius equation, the diffusion coefficient is related to the diffusion activation energy *E*_a_ as *D*(*T*) = *D*_0_exp­(−*E*_a_/*k*_B_ *T*), where *D*_0_ is the pre-exponential factor (also called the frequency factor) and *k*_B_ is the Boltzmann constant. In the liquid state, ln *D* shows a linear relationship with 1000/*T*, with the slope corresponding to the activation energy *E*_a_. Linear fits of pretransition liquid-state dynamics (Figure 4e–f) reveal fundamental differences in molecular mobility across phases. BL-ice I and BL-VHDI exhibit nearly constant diffusion activation energies of 23.94 and 26.33 kJ/mol, respectively, typical of Arrhenius-type behavior in homogeneous liquids. However, BL-PHI exhibits a striking deviation from typical Arrhenius-type behavior in homogeneous liquids, as its diffusion activation energy increases significantly with decreasing temperature, rising from 18.95 to 33.19 kJ/mol. This sharp increase impedes molecular diffusion, dramatically slowing the ice formation process. The impact is striking: while BL-ice I crystallizes within 10 ns and BL-VHDI within 55 ns, BL-PHI’s phase transition requires a microsecond time scale—nearly 2 orders of magnitude slower. This drastic slowdown stems from two key constraints. First, the sharp increase in activation energy significantly impedes molecular rearrangement, leading to a substantial diffusion barrier. Second, BL-PHI’s staggered interlayer stacking demands precise, cooperative molecular motion to establish its hybrid hydrogen-bond network. Unlike BL-ice I and BL-VHDI, where individual molecules can sequentially integrate into AA-stacked structures, BL-PHI requires synchronized adjustments across both layers, further hindering nucleation and growth. These coupled constraints make BL-PHI a “frustrated transient” phase thermodynamically stable within its pressure range but kinetically accessible only under narrowly sustained nonequilibrium conditions.

To examine the dynamic stability of BL-PHI, we perform first-principles density functional theory (DFT) calculations using the VASP package. The optimized structure of BL-PHI, based on the nonlocal-dispersion corrected functional vdw-DF2, is presented in Figure 5a. The BL-PHI unit cell contains 44 water molecules with 22 molecules in each layer. As shown in Figure 5b, BL-PHI exhibits a distinctive 5^5^·6^2^ Archimedean tiling pattern within each monolayer, where pentagonal and hexagonal rings interconnect to form a tessellated network (highlighted in yellow and blue). This geometry arises from the seamless alignment of hexagonal rings along their edges (purple and green markers), creating a stable two-dimensional architecture. In addition, the oxygen atoms in the upper and lower monolayers are stacked in a centrosymmetric manner around a central axis, which is located between the two monolayers and is parallel to the *xy* plane. To further validate its stability, we computed the phonon spectra using density functional perturbation theory (DFPT). No imaginary frequencies are found across the entire Brillouin zone, further confirming the dynamic stability of BL-PHI (Figure 5c). The phonon spectra also reveal peaks corresponding to OH bending (around 1760 cm^–1^) and OH stretching (around 3400 cm^–1^) along with low-frequency collective motion regions.

**Figure 5:**
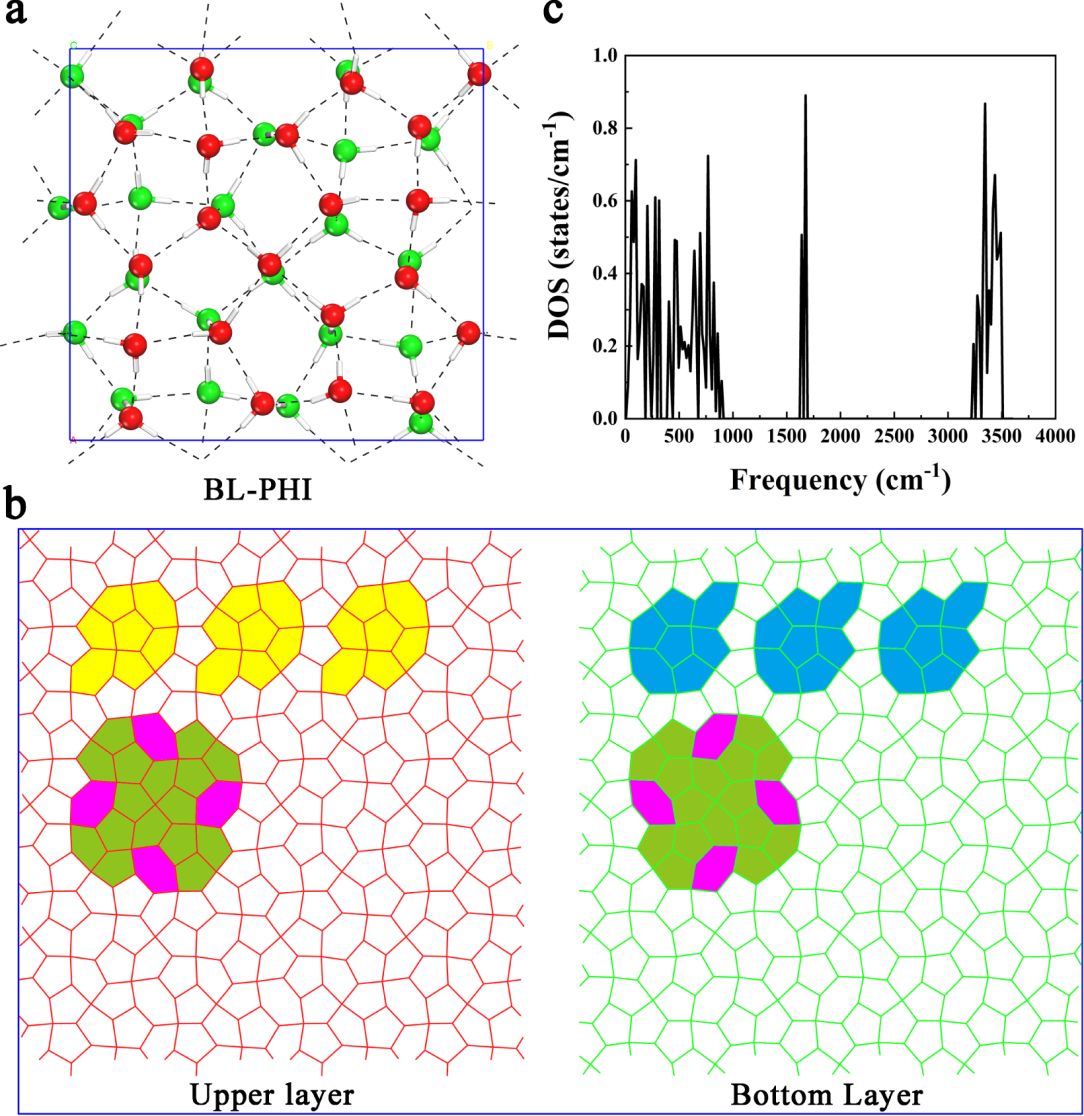
(a) The unit cell of BL-PHI (*L*_*x*_ = 13.06 Å and *L*_*y*_ = 13.78 Å) was optimized using a DFT method. (b) The top views of the upper layer (left) and lower layer (right) of a 4 × 4 supercell. (c) Computed phonon density of states of BL-PHI using the DFPT method implemented in the VASP package.

To extend spatiotemporal sampling, we conduct additional MD simulations using the MLP developed by Jiang et al. This MLP was trained for both monolayer and multilayer water/ice in 6–8 Å nanoslits^17^ and thus differs from monolayer-specific MLPs (e.g., Kapil et al. and Lin et al.) trained for water in monolayer confinement (<6.5 Å).^25,26^ Our MLP-based MD simulations demonstrate that the BL-PHI phase remains structurally stable over a wide lateral pressure range of 400 to 900 MPa at 150 K, as the structure appears to be highly robust for at least 5 ns (see Movies S1–S4). These results are consistent with those from classical MD simulations based on the TIP4P/2005 model, providing additional support for the robustness of the BL-PHI phase.

Furthermore, we carried out free-energy calculations for the relevant phases—BL-ice I, BL-PHI, and BL-VHDI—using the thermodynamic integration (TI) method. The computed Gibbs free energies provide a clear thermodynamic perspective of the phase changes with increasing lateral pressure. As shown in Figure S5, BL-ice I is thermodynamically stable at 300 MPa. As the pressure increases, the free energy of BL-PHI rises more gradually compared to BL-ice I, resulting in a crossing point at around 500 MPa, where BL-PHI becomes the stable phase. Similarly, the BL-PHI phase remains thermodynamically stable until approximately 1400 MPa, beyond which the free energy of BL-VHDI becomes lower, suggesting a transition to the high-density phase. These free-energy results show a pressure-driven sequence of phase stability: BL-ice I → BL-PHI → BL-VHDI, and this sequence confirms that BL-PHI is thermodynamically stable in the intermediate pressure regime, despite its nucleation being kinetically hindered, as shown in our long-time scale MD simulations.

Based on the molecular dynamics (MD) simulations, we construct a lateral pressure versus temperature semiquantitative phase diagram for confined water in a nanoslit with a width of *h* = 8.6 Å. The resulting phase diagram, as shown in Figure 6, provides an intuitive overview of the three ice phases and the bilayer liquid water. In this diagram, the boundaries between liquid and solid phases are defined by transition temperatures at various lateral pressures. Notably, the freezing points (solid color markers) are consistently lower than the melting points (hollow color markers), a characteristic feature of supercooled water behavior. At lateral pressures ranging from 0.1 to 350 MPa, liquid water transforms into BL-ice I phase. Between 400 and 900 MPa, the novel BL-PHI phase forms. At pressures above 1.0 GPa and up to 1.5 GPa, the BL-VHDI phase emerges, consistent with our previous findings. During cooling, the freezing temperature of BL-ice I decreases with increasing pressure, from 270 K at 0.1 MPa to 200 K at 350 MPa. In contrast, for BL-PHI, the freezing temperature initially increases before decreasing, rising from 200 K at 400–450 MPa to 220 K at 600–750 MPa and then dropping to 210 K at 800–900 MPa. A similar trend is observed for BL-VHDI, where the freezing temperature increases from 210 K at 1 GPa to 240 K and then decreases to 230 K at 1.4–1.5 GPa. During heating, the melting temperature of BL-ice I reaches a maximum of 290 K at 50 MPa. For BL-VHDI, the melting temperature rises to 300 K with increasing pressure and then levels off at this value for pressures between 1.2 and 1.5 GPa. For BL-PHI, the melting temperature first increases and then decreases with pressure, reaching a maximum of 260 K at 550–850 MPa.

**Figure 6:**
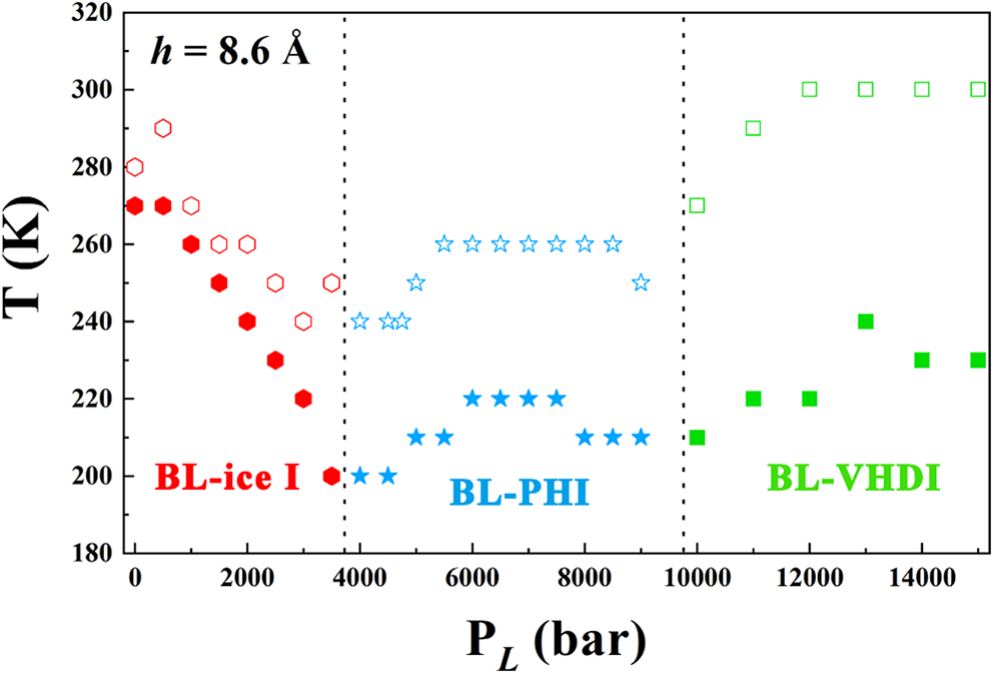
A schematic lateral pressure–temperature phase diagram shows the location of three stable bilayer ice phases (BL-ice I, BL-PHI, BL-VHDI, and BL liquid water). The solid colored points represent the freezing line, and the hollow colored points represent the melting line.

The previous studies have demonstrated that even small changes in pore width can significantly alter the arrangement of water molecules and their hydrogen bonding network, thereby influencing the resulting ice phases. To further explore the effects of geometric confinement, we systematically simulated the behavior of the system under different slit pore widths (ranging from 0.86 to 0.98 nm) and various pressure conditions. The results include representative structures at pore widths of 0.90, 0.94, and 0.98 nm, as shown in Figures S6 and S7. At these pore sizes, we observed AA-stacked bilayer 2D ice structures, as well as a three-layer ice structure, all of which are consistent with those reported in previous studies.

## Conclusions

In this study, we perform extensive molecular dynamics simulations to explore the phase behavior of water confined between smooth walls with a separation of *h* = 8.6 Å. Among the three observed bilayer crystalline 2D ices—BL-ice I, BL-VHDI, and the new BL-PHI structure—BL-PHI stands out due to its unique structural and kinetic characteristics. Characterized by interlocked pentagonal and hexagonal rings, BL-PHI emerges at intermediate to high pressures (400 to 900 MPa). Most notably, BL-PHI exhibits a significantly higher diffusion activation energy prior to the freezing, compared to BL-ice I and BL-VHDI, and hence drastically lower molecular mobility, leading to an exceptionally prolonged phase transition. Unlike BL-ice I and BL-VHDI, which crystallize within nanoseconds, the transition to BL-PHI requires microsecond time scale—nearly 2 orders of magnitude slower. This pronounced kinetic constraint underscores the distinct nature of BL-PHI, setting it apart from other bilayer ices and highlighting its unique role in the complex phase behavior of confined water. Furthermore, our MLP-based MD simulations and free-energy calculations show that the BL-PHI phase is structurally stable at 150 K over a wide lateral pressure range (400–900 MPa) and exhibits thermodynamic stability in the intermediate pressure regime. Although its nucleation is kinetically hindered, as shown by long-time scale MD simulations, BL-PHI is identified as a thermodynamic stable phase rather than a metastable one. Finally, the transition temperatures of the bilayer ices are pressure-dependent. These findings provide deeper insights into the complex phase behavior of water under nanoscale confinement; contribute to a better understanding of water’s behavior in confined environments; and offer valuable implications for applications in nanotechnology, materials science, and environmental science.

## Supplementary Material











## Notes

Details of density functional theory (DFT) computations, the machine learning potential (MLP) molecular dynamics simulations and Gibbs free energy calculation; the snapshots of BL-ice I and BL-VHDI; the potential energy (U) with temperature (T) during the heating process; the in-plane oxygen-oxygen RDFs of the 2D ice system at 200 K; the MSDs at different temperatures; Gibbs free energy of solid phases was computed via thermodynamic integration at 50 K across various pressures; the snapshots of inherent structures at pore widths of 0.90, 0.94, and 0.98 nm (PDF)
Simulation trajectory of the BL-PHI bilayer at 150 K and 400 MPa using a machine learning potential (MP4)
Simulation trajectory of the bottom layer of BL-PHI at 150 K and 400 MPa using a machine learning potential (MLP) (MP4)
Simulation trajectory of the BL-PHI bilayer at 150 K and 900 MPa using a machine learning potential (MLP) (MP4)
Simulation trajectory of the bottom layer of BL-PHI at 150 K and 900 MPa using a S8 machine learning potential (MLP) (MP4)
