# Supplementary material for: Microsecond-Scale Molecular Dynamics Simulation of Phase Transition of a Bilayer Ice: Kinetic Constraints in Confined Water
Source: J Phys Chem B. 2025 Jun 9;129(24):5989–97. doi: 10.1021/acs.jpcb.5c01346 (PMC12183707; doi:10.1021/acs.jpcb.5c01346)
Supplement: Supplementary file 1 [file jp5c01346_si_001.pdf]

Supporting Information

Microsecond-Scale Molecular Dynamics Simulation of Phase Transition of a Bilayer Ice: Kinetic Constraints in Confined Water

Weiduo Zhu[a], Yiyao Li[c], Haidi Wang[a], Zhao Chen[a], Xiaofeng Liu[a], Zhongjun Li[a], Wenhui Zhao,[b,*] and Xiao Cheng Zeng [c,d*]

[a]*Department of Physics, Hefei University of Technology, Hefei, Anhui 230009, China*

[b]*Department of Physics, Ningbo University, Ningbo, Zhejiang 315211, China*

[c]*Department of Materials Science & Engineering, City University of Hong Kong, Kowloon 999077, Hong Kong*

[d]*Department of Chemistry, University of Nebraska-Lincoln, Lincoln, Nebraska 68588, USA*

[*]*Authors to whom correspondence should be addressed: zhaowenhui@nbu.edu.cn, xzeng26@cityu.edu.hk*

Density Functional Theory (DFT) Computations

The nonlocal dispersion corrected functional vdW-DF2 and the PAW potentials were employed in the DFT computation for the relaxation of BL-PHI. The energy cutoff of the plane-wave was taken as 700 eV, and the k-point grids were sampled with a uniform spacing of $2\pi \times 0.025$ Å^{-1}.[S1, S2] All the DFT calculations were carried out in the VASP 6.4 software package.[S3] For computing the phonon spectra, the DFPT (density functional perturbation theory) method was selected, a method implemented in the Phonopy program associated with the VASP software package.[S4]

Machine Learning Potential (MLP) Molecular Dynamics Simulations

Molecular dynamics simulations are performed to validate the stability of BL-PHI phase using MLP developed by Jiang *et al.*[S5] This MLP is trained via the n2p2[S6] program, which is based on Behler Parrinello neural network framework[S7]. The simulations are conducted through the n2p2 interface with LAMMPS package.[S8] To ensure consistency with prior studies, the hydrophobic surface setup replicated the geometry and force field parameters used in GROMACS-based simulations. The simulation cell, constructed as a 3×3×1 supercell of the optimized structure of BL-PHI with, containing 396 water molecules with lateral dimensions of 40 Å in x and y directions. Initial relaxation is conducted under under isothermal-isochoric (NVT) ensemble for 0.5ns to equilibrate atomic positions. Subsequently, MD simulations in isothermal/isobaric-lateral-pressure (NP_LT) ensemble are performed for 5 ns to probe phase stability of BL-PHI phase under controlled lateral stress conditions.

Gibbs Free Energy Calculation

The Gibbs free energy of the three bilayer ices is computed using thermodynamic integration, a method that combines the free energy of an Einstein crystal with the reversible work required to transform the crystal into the target solid. The Einstein crystal approximation treats atoms as independent three-dimensional quantum

harmonic oscillators, neglecting interatomic interactions. The Gibbs free energy G is expressed as: $G = F + PV$ where P is the lateral pressure, V is the nanoslit volume, and F is the Helmholtz free energy. The latter is calculated as: $F = F_{Einstein} + \overline{W^{irr}}$ Here, $F_{Einstein}$ denotes the Helmholtz free energy of the Einstein crystal reference state, and $\overline{W^{irr}}$ represents the reversible work required to transition between the Einstein crystal and the solid.

The spring constant k for the Einstein crystal is determined via the equipartition theorem:

$$\frac{1}{2}k\langle(\Delta r)^2\rangle = \frac{3}{2}k_B T$$

yielding:

$$k = \frac{3k_B T}{\langle(\Delta r)^2\rangle}$$

where $\langle(\Delta r)^2\rangle$ is the mean-squared displacement of all atoms, T is the temperature, and k_B is the Boltzmann constant.

The reversible work $\overline{W^{irr}}$ is evaluated along the Frenkel–Ladd path using:

$$\overline{W^{irr}} = \int_0^1 \langle\frac{\partial H(\lambda)}{\partial \lambda}\rangle_\lambda \, d\lambda$$

where the hybrid Hamiltonian $H(\lambda)$ is defined as: $H(\lambda) = \lambda H_{solid} + (1-\lambda)H_{\mathrm{Einstein}}$. The scaling parameter λ changes from 0 to 1 along the simulation time. Herein, the following switching function is chosen to control the value of λ during scanning Frenkel–Ladd path:

$$\lambda(\tau) = \tau^5(70\tau^4 - 315\tau^3 + 540\tau^2 - 420\tau + 126)$$

with $\tau = \frac{t}{t_s}$, where t is the elapsed time and t_s is the total switching duration. In calculations, switching procedure cost 200000 MD steps, and the simulation system is equilibrated for 50000 steps before initiating the switching procedure. Both forward (λ from 0 to 1) and backward (λ from 1 to 0) scaling are proceeded to ensure accuracy of reversible work[S9].

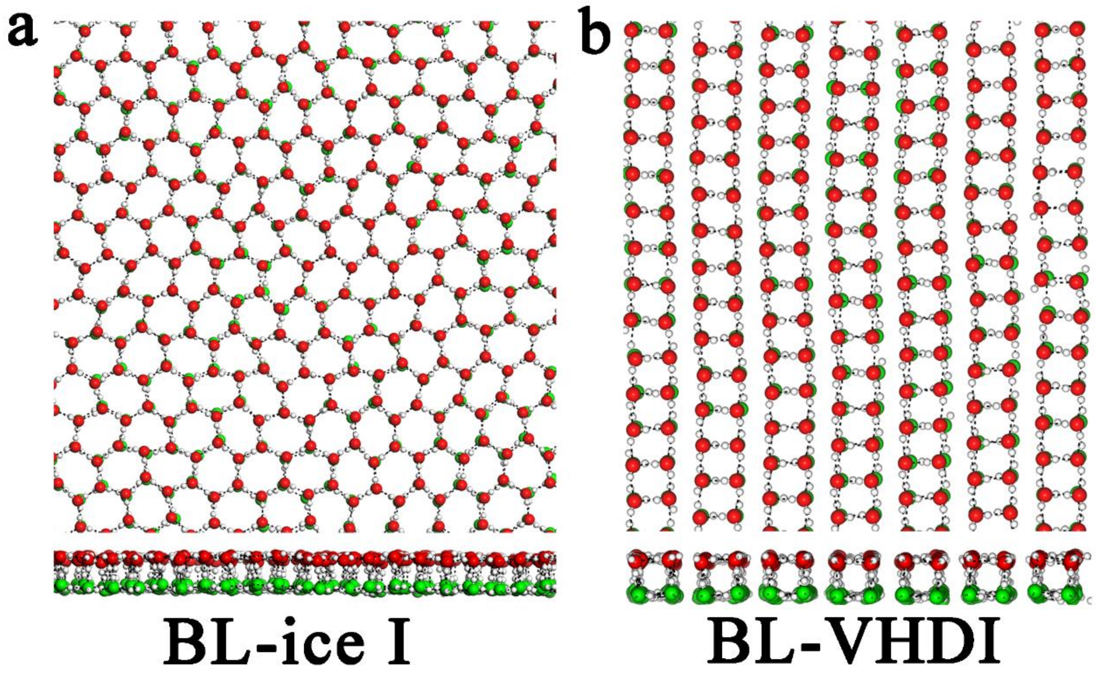

Figure S1. Top view (upper) and side view (lower) of snapshots of inherent structures of (a) BL-ice I at 200 K and 250 MPa, and (b) BL-VHDI at 200 K and 1.2 GPa, formed between two hydrophobic walls with separation h = 8.6 Å. The red spheres are oxygen atoms in the upper layer and the green spheres are oxygen atoms in the lower layer. The white spheres are hydrogen atoms and black dot lines represent the hydrogen bonds.

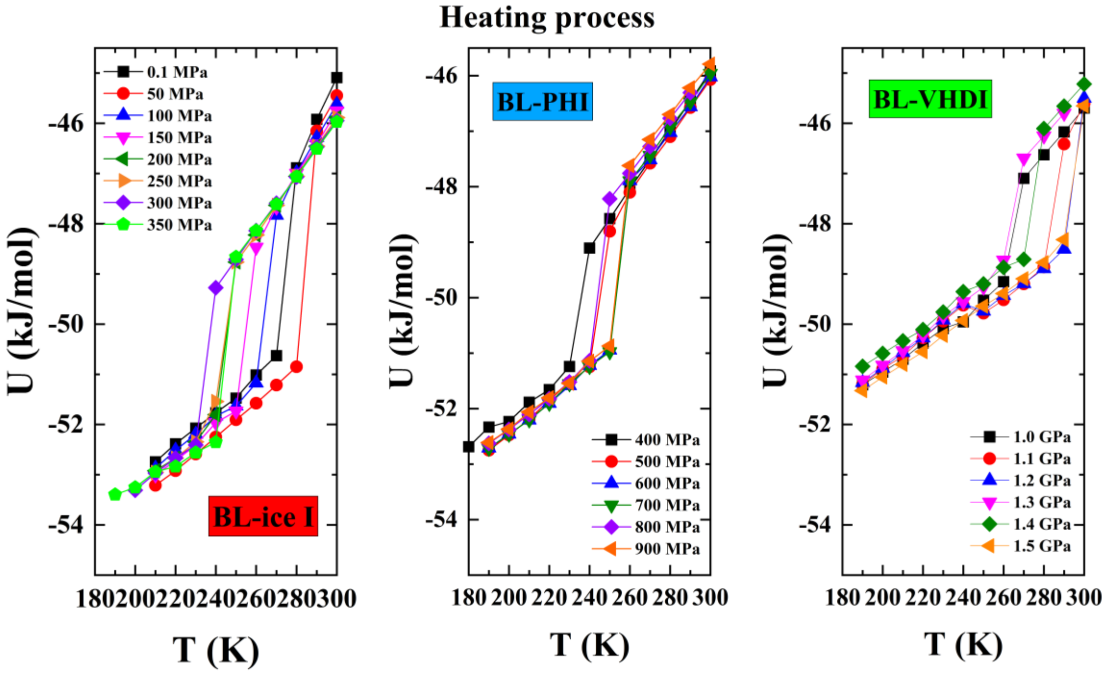

Figure S2. Variation of potential energy (U) with temperature (T) during the heating process at different pressures. (a) lateral pressure (P_L) from 0.1 MPa to 350 MPa for BL-ice I. (b) Lateral pressure (P_L) from 400 MPa to 900 MPa for BL-PHI. (c) Lateral pressure (P_L) from 1.0 GPa to 1.5 GPa for BL-VHDI.

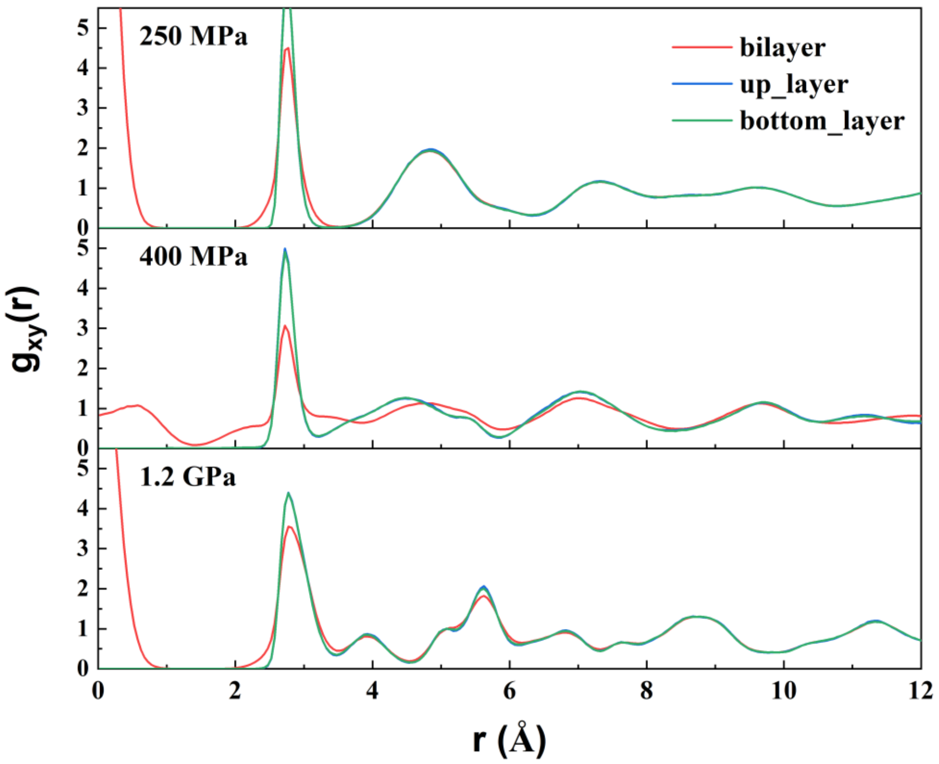

Figure S3. The in-plane oxygen-oxygen radial distribution functions (RDFs) of the 2D ice system at 200 K under varying lateral pressure (P_L) conditions. The red curve denotes the combined bilayer structure, while the blue and green curves separately characterize the upper and lower layers.

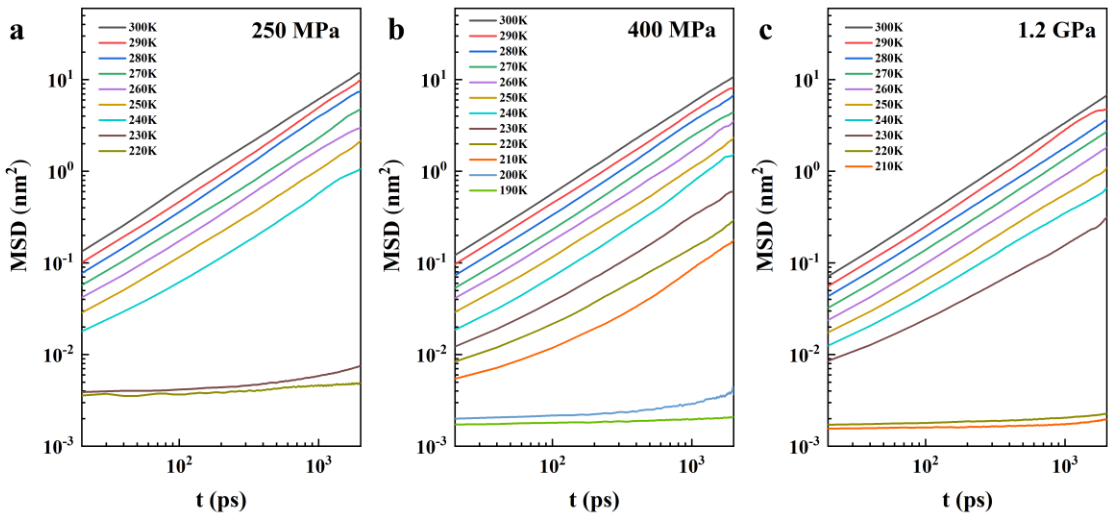

Figure S4. Variation of MSD over time at different temperatures and lateral pressures of (a) 250 MPa, (b) 400MPa, and (c) 1.2 GPa.

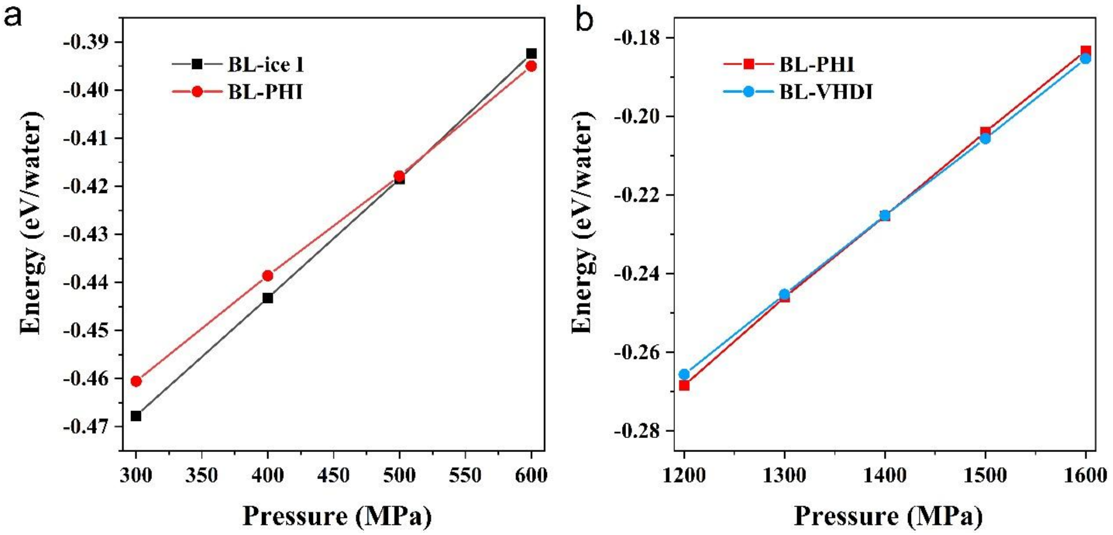

Figure S5. Gibbs free energy of solid phases computed via thermodynamic integration, for the solid at 50 K and different pressures: (a) BL-ice I vs. BL-PHI (300–600 MPa); (b) BL-PHI vs. BL-VHDI (1200–1600 MPa).

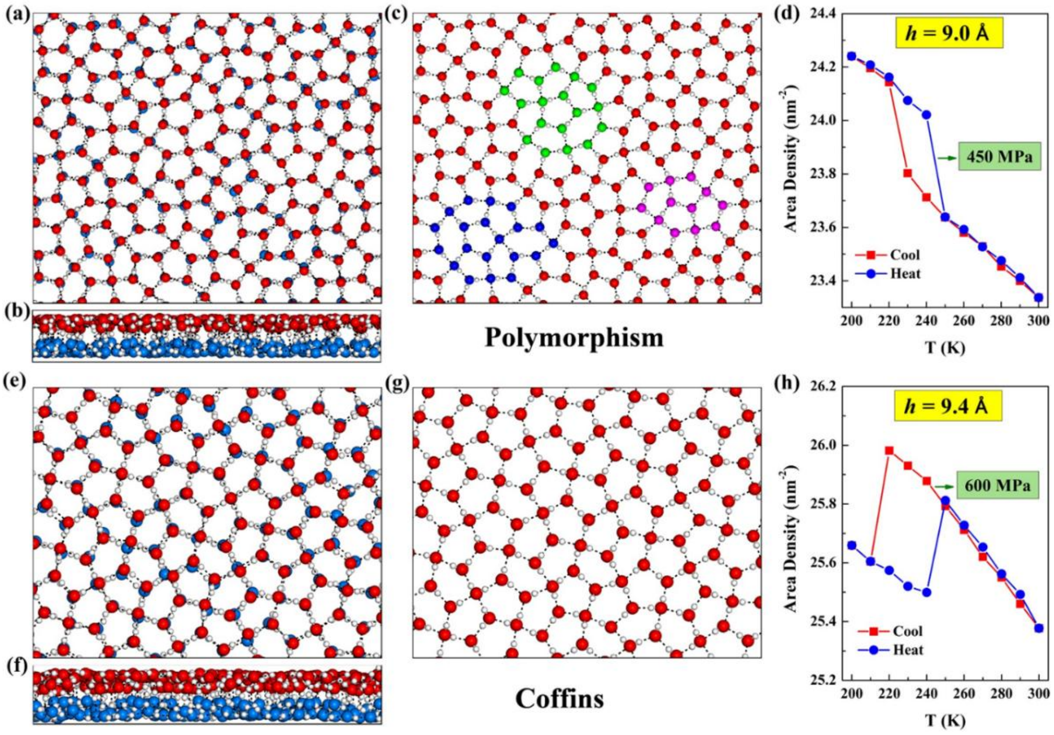

Figure S6. (a) Top view and (b) side view of snapshots showing the inherent structures of the polymorphic phase at 220 K and 450 MPa. The ice is confined between two hydrophobic walls with a separation distance of h = 9.0 Å. (c) Top view of the polymorphic phase highlighting the upper monolayer. (d) Area density versus temperature (T) during the cooling process. (e) Top view and (f) side view of snapshots showing the inherent structures of Coffins at 210 K and 600 MPa, confined

between two hydrophobic walls with a separation distance of h = 9.4 Å. (g) Top view of Coffins highlighting the upper monolayer. (h) Area density versus temperature (T) during the cooling process.

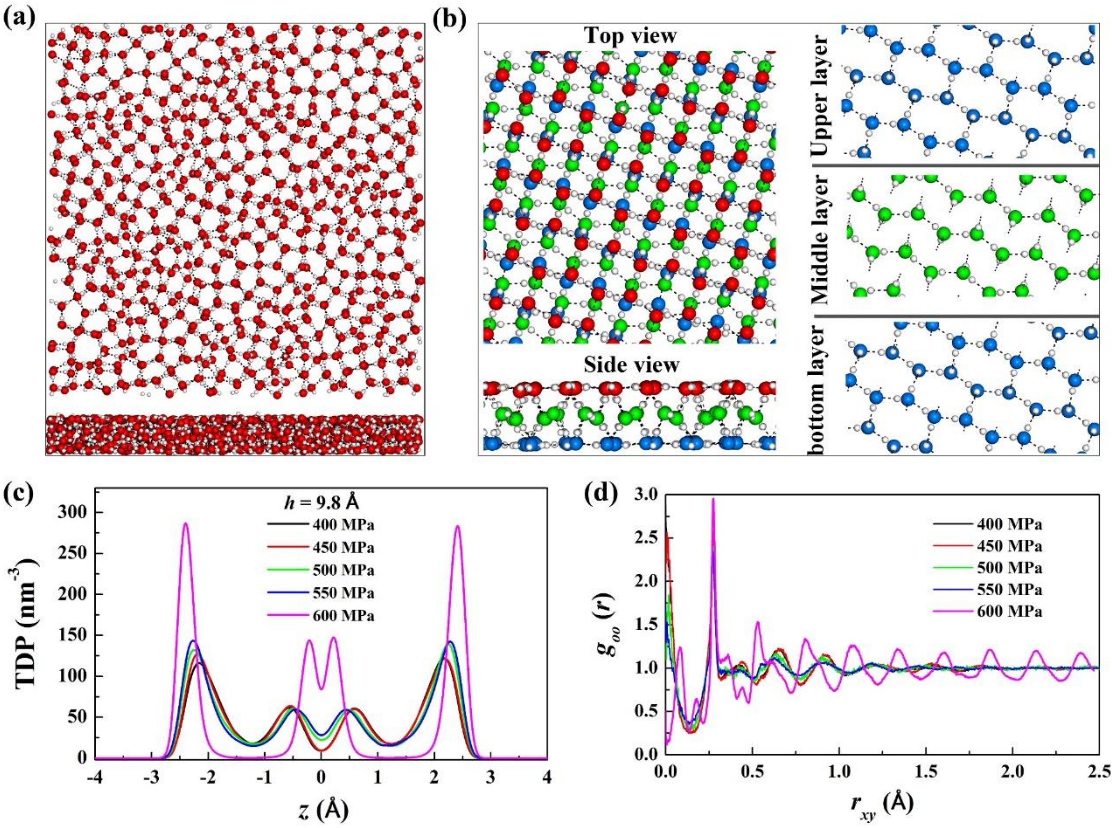

Figure S7. (a) Top and side views of snapshots showing the inherent structures of amorphous ice at 200 K and 550 MPa, confined between two hydrophobic walls with a separation distance of h = 9.8 Å. (b) Top and side views of snapshots showing the inherent structures of trilayer ice at 200 K and 600 MPa. (c) Transverse density profiles (TDPs) of nanoconfined ices at 200 K under different pressures. (d) Oxygen–oxygen radial distribution functions (RDFs) at 200 K under different pressures.

Table S1. The BL-PHI phase was identified through simulations employing the TIP4P/ice water model under different pressure conditions, within a nanoconfinement of 8.6 Å in width.

**Pressure / MPa**	**400**	**500**	**600**	**700**	**800**	**900**
**Temperature / K**	210	220	220	220	220	220

Movie S1. Simulation trajectory of BL-PHI bilayer at 150 K and 400 MPa using a machine learning potential (MLP). (BL_PHI_400MPa_Bilayer.mp4).

Movie S2. Simulation trajectory of the bottom layer of BL-PHI at 150 K and 400 MPa using a machine learning potential (MLP). (BL_PHI_400MPa_BottomLayer.mp4)

Movie S3. Simulation trajectory of BL-PHI bilayer at 150 K and 900 MPa using a machine learning

potential (MLP). (BL_PHI_900MPa_Bilayer.mp4).

Movie S4. Simulation trajectory of the bottom layer of BL-PHI at 150 K and 900 MPa using a machine learning potential (MLP). (BL_PHI_900MPa_BottomLayer.mp4)

Supporting References

(S1) Lee, K.; Murray, É. D.; Kong, L.; Lundqvist, B. I.; Langreth, D. C. Higher-Accuracy van der Waals Density Functional. *Phys. Rev. B* **2010**, *82* (8), 081101.

(S2) Kresse, G.; Joubert, D. From Ultrasoft Pseudopotentials to the Projector Augmented-Wave Method. *Phys. Rev. B* **1999**, *59* (3), 1758-1775.

(S3) Kresse, G.; Furthmüller, J. Efficient Iterative Schemes for ab initio Total-Energy Calculations using a Plane-Wave Basis Set. *Phys. Rev. B* **1996**, *54* (16), 11169-11186.

(S4) Baroni, S.; de Gironcoli, S.; Dal Corso, A.; Giannozzi, P. Phonons and Related Crystal Properties from Density-Functional Perturbation Theory. *Rev. Mod. Phys.* **2001**, *73* (2), 515-562.

(S5) Jiang, J.; Gao, Y.; Li, L.; Liu, Y.; Zhu, W.; Zhu, C.; Francisco, J. S.; Zeng, X. C. Rich Proton Dynamics and Phase Behaviours of Nanoconfined Ices. *Nat. Phys*. **2024**, 20 (3), 456–464.

(S6) Singraber, A.; Behler, J.; Dellago, C. Library-Based LAMMPS Implementation of High-Dimensional Neural Network Potentials. *J. Chem. Theory Comput.* **2019**, 15 (3), 1827–1840.

(S7) Behler, J.; Parrinello, M. Generalized Neural-Network Representation of High-Dimensional Potential-Energy Surfaces. *Phys. Rev. Lett*. **2007**, 98 (14), 146401.

(S8) Thompson, A. P.; Aktulga, H. M.; Berger, R.; Bolintineanu, D. S.; Brown, W. M.; Crozier, P. S.; in 't Veld, P. J.; Kohlmeyer, A.; Moore, S. G.; Nguyen, T. D.; *et al*. LAMMPS - a Flexible Simulation Tool for Particle-Based Materials Modeling at the Atomic, Meso, and Continuum Scales. *Comput. Phys. Commun*. **2022**, 271, 108171.

(S9) Freitas, R.; Asta, M.; de Koning, M. Nonequilibrium Free-Energy Calculation of Solids Using LAMMPS. *Comput. Mater. Sci.* **2016**, 112, 333–341.
